# SAP130 released by damaged tubule drives necroinflammation via miRNA-219c/Mincle signaling in acute kidney injury

**DOI:** 10.1038/s41419-021-04131-7

**Published:** 2021-09-23

**Authors:** Lin-Li Lv, Cui Wang, Zuo-Lin Li, Jing-Yuan Cao, Xin Zhong, Ye Feng, Jun Chen, Tao-Tao Tang, Hai-Feng Ni, Qiu-Li Wu, Bin Wang, Hui-Yao Lan, Bi-Cheng Liu

**Affiliations:** 1 Institute of Nephrology, Zhongda Hospital, Southeast University School of Medicine, Nanjing, Jiangsu Province 210009 China; 2 Department of Medicine & Therapeutics, Li Ka Shing Institute of Health Sciences, The Chinese University of Hong Kong, Hong Kong Special Administrative Region, Hong Kong, China

**Keywords:** Cell death and immune response, Acute kidney injury

## Abstract

Tubules injury and immune cell activation are the common pathogenic mechanisms in acute kidney injury (AKI). However, the exact modes of immune cell activation following tubule damage are not fully understood. Here we uncovered that the release of cytoplasmic spliceosome associated protein 130 (SAP130) from the damaged tubular cells mediated necroinflammation by triggering macrophage activation via miRNA-219c(miR-219c)/Mincle-dependent mechanism in unilateral ureteral obstruction (UUO) and cisplatin-induced AKI mouse models, and in patients with acute tubule necrosis (ATN). In the AKI kidneys, we found that Mincle expression was tightly correlated to the necrotic tubular epithelial cells (TECs) with higher expression of SAP130, a damaged associated molecule pattern (DAMP), suggesting that SAP130 released from damaged tubular cells may trigger macrophage activation and necroinflammation. This was confirmed in vivo in which administration of SAP130-rich supernatant from dead TECs or recombinant SAP130 promoted Mincle expression and macrophage accumulation which became worsen with profound tubulointerstitial inflammation in LPS-primed Mincle WT mice but not in Mincle deficient mice. Further studies identified that Mincle was negatively regulated via miR-219c-3p in macrophages as miR-219c-3p bound Mincle 3′-UTR to inhibit Mincle translation. Besides, lentivirus-mediated renal miR-219c-3p overexpression blunted Mincle and proinflammatory cytokine expression as well as macrophage infiltration in the inflamed kidney of UUO mice. In conclusion, SAP130 is released by damaged tubules which elicit Mincle activation on macrophages and renal necroinflammation via the miR-219c-3p-dependent mechanism. Results from this study suggest that targeting miR-219c-3p/Mincle signaling may represent a novel therapy for AKI.

## Background

Acute kidney injury (AKI) is characterized by a complex interacting process including cell death, infiltration of immune cells, and the following cellular maladaptive repair processes [1]. Injury and death of tubular epithelial cells (TECs) is the primary pathological characteristic of AKI [2, 3], and necroinflammation is formed as an autoamplification loop between tubular cell death and interstitial inflammation, leading to the exacerbation of renal injury [2]. Macrophages are considered to be key inflammatory cells that participated in the progression and repair of AKI [4]. However, the exact modes of macrophage activation following tubular damage are not fully understood.

Damage-associated molecular patterns (DAMPs) are endogenous molecules released upon cellular activation, stress, or damage and can induce the activation of inflammatory pathways during sterile inflammation [5]. Accumulating evidence suggested that a variety of DAMPs released from dead cells triggered immune responses and contributed to the pathogenesis of kidney disease [6–9]. DAMPs initiate kidney inflammation through recognizing their respective pattern recognition receptors (PRRs), including Toll-like receptors, purinergic receptors, or the NLRP3 inflammasome [6]. DAMPs could be derived from the subcellular origin, i.e. cytoplasm, endoplasmic reticulum, nucleus, and mitochondria. However, which DAMPs material is released from damaged TECs and promotes macrophage activation via specific PRRs remained to be explored.

Macrophage inducible C-type lectin (Mincle) is a member of the C-type lectin receptor family and is encoded by *Clec4e*. Mincle was shown to be a crucial PRR in sensing pathogens as well as endogenous ligands [10, 11]. In recent years, Mincle is largely described as a sensor to recognize glycolipids from pathogens, it also binds to DAMPs released by dead cells such as spliceosome-associated protein 130 (SAP130), β-glucosylceramide (β-GlcCer), cholesterol sulfate, and crystals [10, 12–14]. Importantly, Mincle activation through recognizing SAP130 contributed to the ethanol-induced liver injury, traumatic brain injury, and pancreatic ductal adenocarcinoma progression [14–16]. Our previous studies showed that rapid induction of Mincle after kidney injury which played a critical role in the acute phage of kidney inflammation [17]. However, the mechanism of Mincle activation in AKI remains to be explored. In this study, we aimed to investigate whether SAP130 was released from injured TECs and contributed to Mincle and macrophage activation.

Our data demonstrated that the release of cytoplasmic SAP130 from injured TECs was increased in both the AKI mice model and clinic patients with acute tubule necrosis (ATN). Further study demonstrated that SAP130 originated from damaged tubules promoted necroinflammation by triggering macrophage activation via miRNA-219c (miR-219c)/Mincle-dependent mechanism. Thus, this study identified a novel model of macrophage activation caused by damaged tubules which may represent a new therapeutic target for AKI.

## Results

### SAP130 expression in injured tubule correlated with mincle expression

**Fig. 1:**
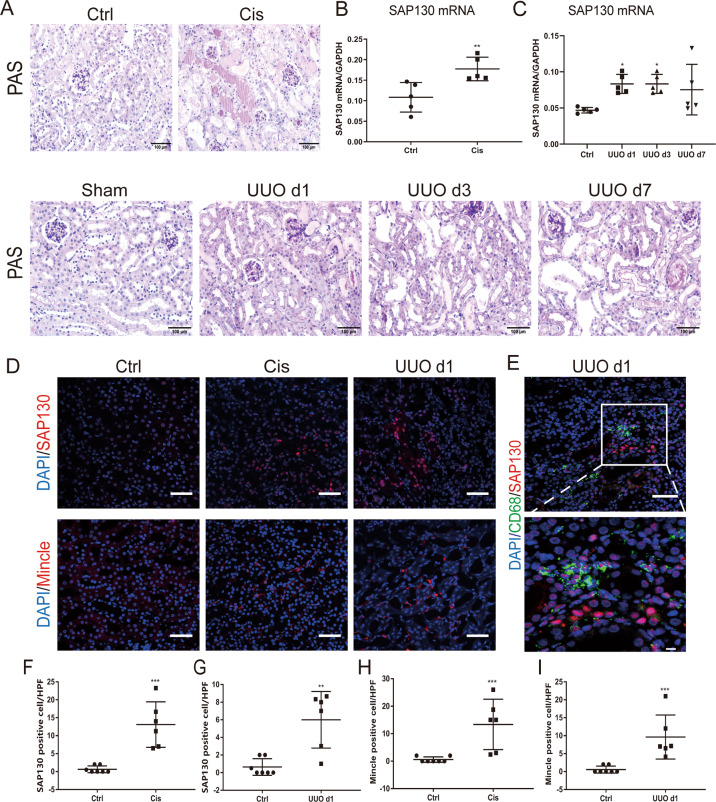
Correlation between SAP130 and Mincle expression in mouse model of acute kidney injury. **A** Acute kidney injury (AKI) was induced by unilateral ureteral obstruction (UUO) and cisplatin injection in mice. Scale bar, 100 μm. **B**–**C** SAP130 expression was increased in AKI mice compared to controls as detected by RT-PCR. **D** Immunofluorescence staining showed increased cytoplasmic SAP130 expression which is evident in tubules of the injured kidney, accompanying with upregulated expression of Mincle. Scale bar, 50 μm. **E** Confocal microscopy showed that CD68^+^ macrophages (green) were surrounded to the area of tubules with strong SAP130 expression (red). Scale bar, 50 μm. **F**–**I** Quantitative analysis of SAP130 and Mincle positive cells. Data were presented as mean ± SD. *p < 0.05, **p < 0.01, ***p < 0.001 compared to Ctrl. Ctrl Control, Cis cisplatin.

We have previously shown that Mincle expression and macrophage accumulation are induced in obstructed kidneys of the UUO model as well as in cisplatin-induced acute kidney injury [17]. To explore the pathological link between acute tubule injury and Mincle activation, SAP130 as the first described endogenous ligand of Mincle was detected in the AKI mice model. We found that SAP130 expression was upregulated significantly in kidneys of both UUO and cisplatin-treated mice compared to normal controls (Fig. 1A–C). By immunofluorescence staining, increased cytoplasmic SAP130 expression was observed in injured TECs in which Mincle was highly co-expressed with profound macrophage accumulation (Fig. 1D–I).

**Fig. 2:**
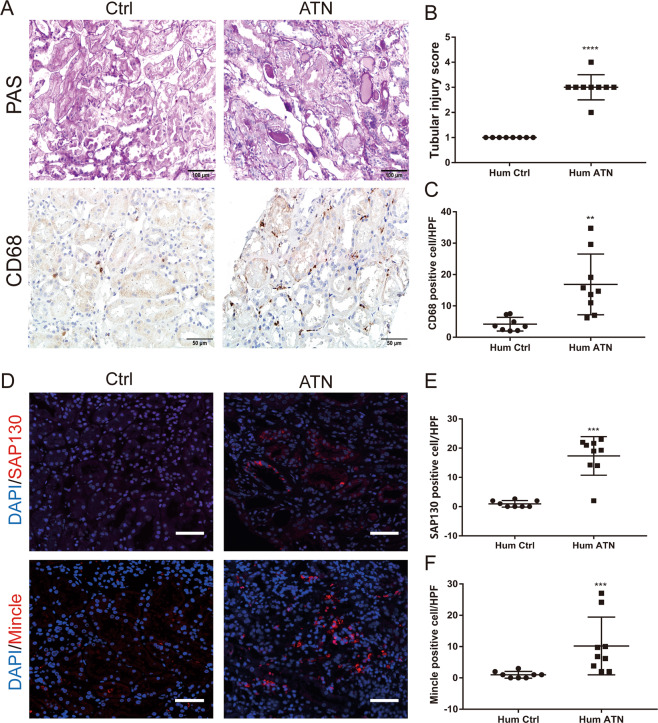
Enhanced SAP130 and Mincle expression in patients with acute tubule necrosis. **A**–**C** Patients with acute tubular necrosis (ATN, *n* = 9) and patients with glomerular minor lesion as controls (Ctrl, *n* = 8) were enrolled. Renal pathology and semiquantitative analysis showed significant tubule injury in ATN patients compared to controls. Scale bar, 100 μm. Macrophages were detected by CD68 immunostaining. Scale bar, 50 μm. **D** Enhanced SAP130 and Mincle expression were detected in ATN patients by immunofluorescence staining. Scale bar, 50 μm. **E**–**F** SAP130 and Mincle positive cells were quantified. Data were presented as mean ± SD. **p < 0.01, ***p < 0.001, and ****p < 0.0001 compared to Ctrl. Ctrl Control, Hum ATN human acute tubular necrosis.

To explore the clinical relevance in human disease, we examined Mincle and SAP130 expression in patients with AKI. Patients with acute tubular necrosis (ATN, *n* = 9) and patients with glomerular minor lesions as controls (Ctrl, *n* = 8) were enrolled. The clinical characteristics of these patients were shown in Table 1. A significant increase in the level of serum creatinine and blood urea nitrogen was present in the ATN group compared to the Ctrl group. Kidney histology of PAS staining showed denudation and flattering of the renal tubular cells, loss of brush border, and intratubular cast formation in ATN patients but absent in control patients (Fig. 2A, B). It was noted that a significantly increased number of CD68 positive macrophages were infiltrated especially around damaged tubules (Fig. 2A, C). Consistent with the findings in UUO and cisplantin-induced AKI mice, SAP130 as well as Mincle was highly expressed in patients with ATN, whereas, a bare expression was observed in patients with a glomerular minor lesion (Fig. 2D–F).

**Table 1:** Clinic characteristics of acute tubular necrosis (ATN) patients and controls.

	Ctrl (*n* = 8)	ATN (*n* = 9)	*P* value
Male/Female	4 /4	4 /5	/
Age	33 ± 4.297	59.67 ± 3.801	***
Scr (umol/L)	59.51 ± 4.972	253.6 ± 59.74	**
BUN (mmol/L)	5.158 ± 0.5043	10.73 ± 2.002	*
UA (umol/L)	334.5 ± 36.37	349.1 ± 48.77	0.8171
24 h proteinuria (g)	5.782 ± 1.317	3.624 ± 1.655	0.3319

*Ctrl* control, patients with glomerular minor lesion were used as controls, *ATN* acute tubular necrosis, *SCr* serum creatinine; *BUN* blood urea nitrogen, *UA* uric acid. *p < 0.05, **p < 0.01, ***p < 0.001 vs. Ctrl.

### SAP130 released from the damaged tubular epithelial cells provoked macrophage activation and renal interstitial inflammation through mincle signaling

**Fig. 3:**
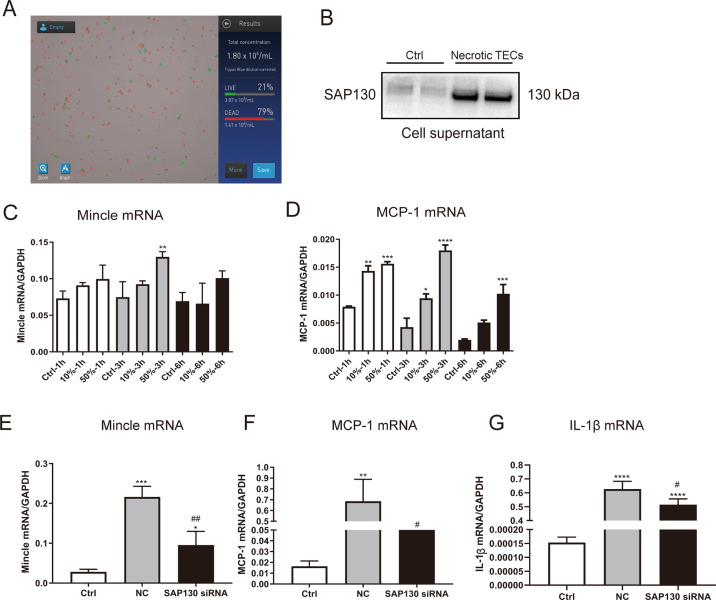
Dead tubules provoke Mincle and proinflammatory cytokine expression in macrophage via SAP130. **A** Dead cells were stained by Trypan blue and detected by Countess™ II FL Automated Cell Counter. **B** SAP130 was detected in the supernatant of dead tubule epithelial cells (TECs) induced by heat stress, while expression was barely detected in normal cells. **C**–**D** Mincle and MCP-1 expression was induced in a dose and time-dependent manner in macrophages treated with SAP130-enriched supernatant of dead TECs. **E**–**G** SAP130 siRNA transfection reversed the proinflammatory effect caused by dead TECs. Data were presented as mean ± SD. *p < 0.05, **p < 0.01, ***p < 0.001, and ****p < 0.0001 compared to Ctrl. ^#^*p* < 0.05, ^##^*p* < 0.01 compared to NC. Ctrl Control, NC Scramble negative control.

We next examined whether SAP130 released from injured TECs can activate Mincle signaling in macrophages. Dead TECs which were confirmed by trypan blue staining (Fig. 3A) were produced by heating and supernatant were collected as reported before [14, 18]. Interestingly, we found an abundant amount of SAP130 released into culture supernatant in TECs suffered from heat stress, while it was hardly detected in the supernatant of normal cells (Fig. 3B). Furthermore, the culture medium from heat-treated TECs caused upregulation of macrophage Mincle and inflammatory cytokine expression in a dose-dependent manner, which was reversed by knocking down SAP130 with siRNA (Fig. 3C–G).

**Fig. 4:**
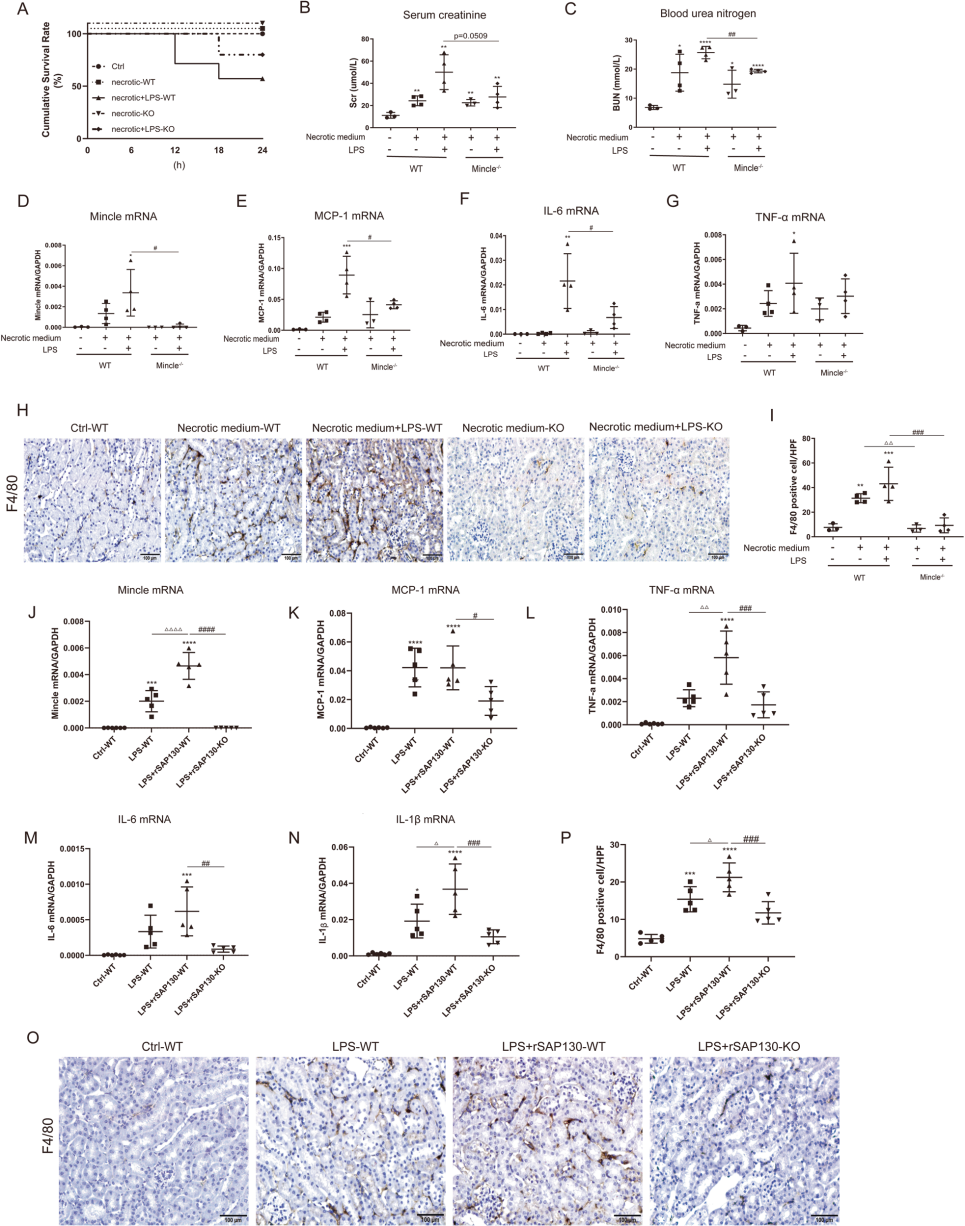
Damaged tubules promoted renal tubulointerstitial inflammation through Mincle signaling. Supernatant from dead TECs was collected and administered to WT and Mincle^−/−^ mice. The inflammatory response was induced by supernatant of dead TECs which was worsen in mice primed with LPS. This synergistic proinflammatory effect was reversed partly in Mincle deficiency mice. **A** Survival curve showed an enhanced survival rate with Mincle deletion in mice administered with necrotic medium primed with LPS. **B**–**C** Upregulation of serum creatinine and blood urea nitrogen were reversed significantly in Mincle^−/−^ mice. **D**–**G** Mincle and inflammatory cytokine expression (MCP-1, IL-6, TNF-α) was upregulated by TECs necrotic medium as detected by RT-PCR. **H**–**I** Macrophage infiltration was detected by F4/80 immunostaining. Recombinant SAP130 was applied to WT and Mincle^−/−^ mice primed with LPS. Scale bar, 100 μm. **J**–**N** Upregulation of Mincle and inflammatory cytokine (MCP-1, TNF-α, IL-6, IL-1β) by rSAP130 was detected by RT-PCR. **O**–**P** Macrophage infiltration was detected by F4/80 immunostaining. Scale bar, 100 μm. Data were presented as mean ± SD. *p < 0.05, **p < 0.01, ***p < 0.001 and ****p < 0.0001 compared to Ctrl; ^#^*p* < 0.05, ^##^*p* < 0.01, ^###^*p* < 0.001, and ^####^*p* < 0.0001; ^△^*p* < 0.05, ^△△^*p* < 0.01, ^△△△△^*p* < 0.0001. Ctrl Control, LPS lipopolysaccharide, WT wild type, KO knockout, rSAP130 recombinant SAP130.

To further demonstrate the SAP130 released from dead TECs can promote renal interstitial inflammation via Mincle, cultured supernatant from dead TECs was administered to mice through intraperitoneal injection. Since SAP130 and LPS synergistically induced more robust inflammatory responses, a group of mice primed with a low dose of LPS was also included as described before [15]. Mincle knockout mice exhibited an increased survival rate compared to the WT group following injection of necrotic medium synergized with low-dose LPS (Fig. 4A). We found that administration of necrotic medium induced renal dysfunction, upregulation of Mincle, and macrophage accumulation in Mincle WT mice, which became worsen in LPS-primed mice, resulting in profound inflammatory cytokine expression (MCP-1, IL-6, TNF-α) and infiltration of F4/80 positive macrophages. In contrast, mice lacking Mincle were protected from SAP130 or the synergistic effect of low LPS plus SAP130-induced necroinflammation in Mincle KO mice (Fig. 4B–I).

In addition, recombinant SAP130 (rSAP130) was administered in vivo to further verify SAP130/Mincle signaling in renal inflammation. Similarly, we observed that administration of rSAP130 in LPS-primed Mincle WT mice promoted Mincle expression and renal interstitial inflammation as indicated by F4/80 positive macrophages accumulation and increased proinflammatory cytokines (MCP-1, TNF-α, IL-6, IL-1β) expression. Impressively, Mincle deletion significantly reversed tubulointerstitial inflammation resulted from rSAP130 exposure (Fig. 4J–P). Taken together, these results suggested that SAP130 from dead TECs was capable of promoting macrophage activation and interstitial inflammation via Mincle signaling.

### Dead tubule promoted mincle expression and proinflammatory macrophage activation via miR-219c-3p

**Fig. 5:**
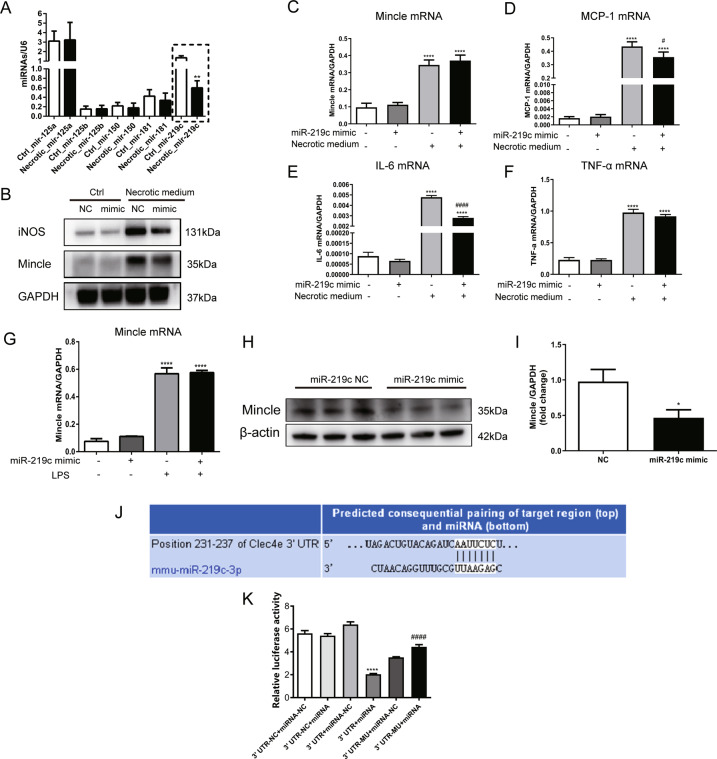
Damaged tubule promoted Mincle expression and proinflammatory macrophage activation through miR-219c-3p. **A** Five miRNAs predicted to bind 3′-UTR of Mincle by bioinformatics analysis were measured by RT-PCR. **p < 0.01 compared to Ctrl. **B** Increased expression of iNOS and Mincle protein induced by LPS were inhibited by miR-219c-3p mimic. **C**–**F** miR-219c-3p mimic transfection reversed partly the upregulation of inflammatory cytokine (MCP-1, IL-6) induced by the necrotic medium of TECs. ****p < 0.0001 compared to groups without treatment of necrotic medium. ^#^*p* < 0.05, ^####^*p* < 0.0001 compared to cells exposed to necrotic medium with negative control of microRNA mimic. **G**–**I** Mincle translation induced by LPS was inhibited by miR-219c-3p mimic without alteration of mRNA level. ****P < 0.0001 compared to groups without LPS treatment. *p < 0.05 compared to NC. **J**–**K** Dual-luciferase reporter gene assay found that Mincle translation was inhibited by miR-219c-3p through binding to the 3′-UTR of Mincle. ****p < 0.0001 compared to 3′ UTR-NC + miRNA, ^####^*p* < 0.0001 compared to 3′UTR + miRNA. Data were presented as mean ± SD. Ctrl Control, NC negative controls, LPS lipopolysaccharide.

Next, the mechanism of Mincle activation in macrophages exposed to dead TECs was further explored. Recent studies found that miRNAs can regulate macrophage activation [19]. Therefore, we postulated that dead TECs might promote Mincle expression via microRNAs. MicroRNAs with target region on 3′-UTR of Minlce were predicted by Targetscan. Forty-five miRNAs with Context + + score percentile above 90 were found, among which miR-181a-5p, miR-125a-3p, miR-150-5p, miR-125b-2-3p and miR-219c-3p were chosen as they are reported to be involved in the activation of macrophages [20–24]. RT-PCR analysis showed that miR-219c-3p was downregulated significantly in macrophages treated with dead cell supernatant, whereas no significant alteration was observed for the other miRNAs (Fig. 5A). Impressively, miR-219c-3p mimic reversed the upregulation of Mincle protein despite no significant change in Mincle mRNA (Fig. 5B, C). Moreover, miR-219c-3p mimic inhibited Mincle-mediated M1 phenotype polarization as demonstrated by decreased expression of iNOS and proinflammatory cytokine (MCP-1, IL-6) (Fig. 5B, D–F). Besides, upregulation of Mincle protein by LPS was also remarkably inhibited by miR-219c-3p mimics, although no significant alteration was observed in the mRNA level (Fig. 5G–I). Those data suggested that dead TECs provoked Mincle expression and proinflammatory macrophage phenotype transition via miR-219c-3p.

Bioinformatics prediction software (TargetScan online analysis, Release 7.2) found that Mincle was the predictive target gene of miR-219c-3p (Fig. 5J). To confirm whether Mincle was inhibited as the direct target gene of miR-219c-3p, a luciferase reporter gene assay was performed. MiR-219c-3p overexpression vector transfection significantly reduced luciferase expression in Mincle-3′UTR transfected cells, but not the Mincle 3′UTR mutant nor the microRNA scrambled-sequence group (miRNA negative control, miRNA-NC) (Fig. 5K). The results confirmed the functional binding of miR-219c-3p in Mincle 3′-UTR and its regulation on Mincle translation.

### MiR-219c-3p overexpression attenuated mincle expression and tubulointerstitial inflammation

**Fig. 6:**
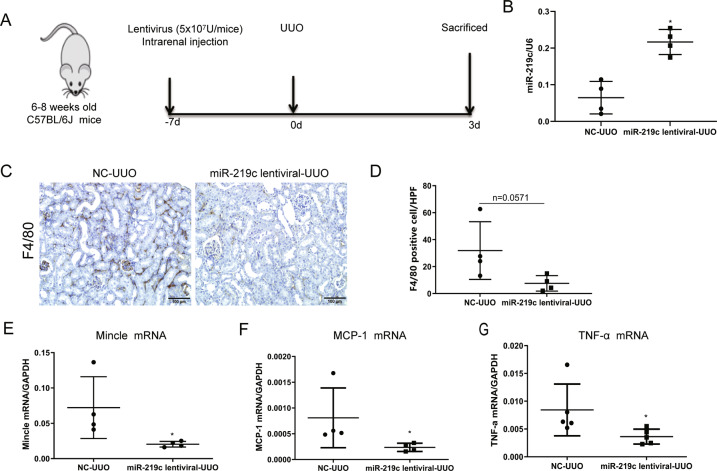
MiR-219c-3p overexpression ameliorated acute kidney inflammation. **A** Schematic overview of study in AKI model induced by UUO mice with miR-219c-3p overexpression via lentiviral vector transfection. **B** Expression of miR-219c-3p in the UUO kidney transfected with miR-219c lentiviral or NC as control. **C**–**D** Representative sections of mice kidney stained for F4/80^+^ macrophages. The number of infiltrated macrophages were quantified. Scale bar, 100 μm. **E**–**G** Mincle, MCP-1, TNF-α was detected by RT-PCR in the kidney which were inhibited by miR-219c-3p overexpression. Data were presented as mean ± SD. *p < 0.05 compared to NC_UUO. NC_UUO, UUO mice treated with empty lentiviral vector as a negative control. MiR-219c lentiviral_UUO, UUO mice treated with miR-219c-3p overexpression via lentiviral transfection.

Next, the regulatory role of miR-219c-3p in Mincle expression was investigated in acute kidney inflammation in vivo. MiR-219c-3p was overexpressed via renal parenchymal injection of lentiviral vectors, and empty lentiviral vectors were introduced as negative control (NC), mice were then subjected to UUO surgery (Fig. 6A). MiR-219c-3p overexpression in mice was confirmed as shown in Fig. 6B. Impressively, immunohistochemistry staining showed that F4/80^+^ macrophages infiltration was decreased in mice with miR-219c-3p overexpression with marginal statistical significance (Fig. 6C, D). Besides, Mincle expression as well as inflammatory cytokines MCP-1, TNF-α in kidney was significantly reduced in mice with miR-219c-3p overexpression, compared to the NC group (Fig. 6E–G). Thus, miR-219c-3p may negatively regulate Mincle signaling and suppress renal inflammation during the acute phase of kidney injury.

## Discussion

Mincle is a C-type lectin receptor that senses cell death and induces the production of inflammatory cytokines to drive the infiltration of inflammatory cells into damaged tissue. Our previous studies demonstrated that Mincle expression is essential for maintaining proinflammatory macrophage phenotype in acute inflammation of kidney disease [17]. However, the mechanism of Mincle activation during kidney injury remains to be clarified.

Firstly, we found that cytoplasmic SAP130 was increased in injured TECs accompanied with Mincle expression in AKI mice as well as in patients with acute tubule necrosis. Similarly, high cytoplasmic SAP130 expression in human pancreatic ductal adenocarcinoma (PDA) was observed which correlated with high RIP1/RIP3 expression, the key regulators of necroptosis [25]. Cell damage results in the release of an array of DAMPs which are related to the activation of innate inflammatory signaling [26]. However, the identity of these endogenous proinflammatory molecules in the kidney is still limited. During ischemic and obstructive injury of the kidney, high mobility group protein B1(HMGB1) nuclear-cytoplasmic translocation and systemic release increased and facilitated M1 macrophage phenotype transition [27, 28]. Inhibition of HMGB1 release may alter macrophage phenotypes and ameliorated UUO-induced renal fibrosis [28]. Moreover, in a recent study reported by Poluzzi et al, soluble biglycan was found as a bimodal DAMP that contributed to recruiting proinflammatory macrophages as well as resolution of inflammation in ischemic AKI [29]. In addition, other kidney-specific DAMPs, such as crystals and uromodulin released by damaged tubules were found [6]. Interestingly, a recent study reported that β-glucosylceramide in combination with free cholesterol were endogenous ligand of Mincle after AKI [30]. The danger molecule SAP130 was the first established endogenous ligand of Mincle [14]. Herein, SAP130 was identified as crucial DAMP to induce macrophage activation in the acute stage of kidney injury. TECs represent the most abundant resident cells of the nephron that are vulnerable to damage in AKI which has been increasingly recognized as the driving force for the progression of kidney disease [2]. Impressively, released SAP130 was predominantly detected in damaged or necrotic tubules which suggested TECs were the primary cellular source of this specific DAMP in the kidney. Previous evidence suggested that SAP130 was released via diverse cell death pathways such as late apoptosis, necrosis [14], and necroptosis [25]. Since various types of cell death such as apoptosis and regulated necrosis could be involved in the pathogenesis of AKI [31, 32], the predominant type of death that is responsible for the SAP130 release needs further investigation.

Secondly, we found that SAP130 from deadly injured TECs was able to trigger Mincle expression and macrophage activation to produce proinflammatory cytokines, resulting in tubular necroinflammation. Macrophage phenotype switch orchestrates the inflammation and repair/regeneration process following AKI. However, to date it is not clear how this phenotype switch is regulated under precise circumstances during sterile inflammation [9]. SAP130, a component of the small nuclear ribonucloprotein, which diffuses out of dying cells is a known endogenous ligand for Mincle [14]. SAP130/Mincle axis participates in the pathogenesis of necroinflammation during tissue damages [14, 33], cancer progression [25], or ischemia/reperfusion [34]. In the early injury phase of AKI, DAMPs released from dying cells drive sterile inflammation and contribute to the necroinflammation and tissue injury [35]. Our data suggested that the SAP130 derived from dead TECs promoted proinflammatory macrophage activation and tubulointerstitial inflammation via the Mincle receptor. Thus, SAP130 in the microenvironment may constitute a crucial component that regulates macrophage phenotype during kidney injury. As an innate immune PRR, Mincle has been reported to mainly express on macrophages, monocytes, neutrophils, and dendritic cells (DCs) [36]. Hence, more observations are required to clarify that whether similarly pathway of SAP130/Mincle in other immune cells may also contribute to necrotic inflammation in AKI.

Thirdly, we found miR-219c-3p is a novel regulator of Mincle expression in macrophages exposed to SAP130. Tanaka et al reported that Mincle is markedly increased in AKI and persisted to induce AKI to chronic kidney disease transition [30]. However, the molecular pathway which induces Mincle expression remains unclear. It is generally recognized that Mincle expression is controlled by NF-IL6 (also called C/EBPb) [37] as well as NF-κB [17] in the acute phase of inflammation. Our data revealed for the first time that Mincle was negatively regulated by miR-219c-3p. Similarly, previous studies showed that microRNAs such as miR-155, miR-223 participated in modulating macrophage polarization via targeting SOCS1, CEBP, and Pknox1, which indicate the crucial role of miRNAs in macrophage phenotype transition [38]. Hence, we demonstrated that the proinflammatory phenotype of macrophages expressing Mincle was regulated by miR-219c-3p during AKI. SAP130 may activate Mincle signaling not only through recognizing its receptor but also inducing the upregulation of Mincle expression.

Thus, SAP130/miR-219c-3p/Mincle axis may provide a new therapeutic target for ameliorating the inflammation response caused by damaged tubules. Current therapy of AKI relies primarily on supportive management [39], however, an effective and specific approach targeting the mechanism of the disease is still lacking. In this study, we found that Mincle activation and renal inflammation during AKI were attenuated significantly by targeting miR-219c-3p. Tremendous endeavor has been made to limit inflammatory diseases via inhibiting regulated cell death [40]. However, interfering with the signaling pathway activated by specific DAMPs could be a rational choice in conditions of established injury. As expected, we found that miR-219c-3p overexpression blunt macrophage infiltration at day 3 after UUO surgery. Besides, Xie et al reported that albumin directly bound to the Mincle receptor, deactivated the downstream pathways which prevented the crosstalk from neuronal necroptosis production SAP130 to launch innate immunity in subarachnoid hemorrhage [41]. Blockade of endogenous ligand SAP130 was also efficient to control the development of multiple sclerosis [42]. However, it should be noted that, in addition to SAP130, damaged kidneys could release other DAMPs during AKI, including β-glucoseramide and cholesterol, which also acts as an endogenous ligand for Mincle [30]. Furthermore, Mincle signaling could be amplified via TLR4 in the context of AKI [17]. Herein, it might be appealing to explore strategies by targeting Mincle signaling for the treatment of AKI.

In conclusion, we demonstrated that SAP130 released from damaged TECs promoted macrophages activation and tubulointerstitial inflammation via Mincle signaling, while miR-219c-3p was identified as a crucial regulator of such process. Hence, the finding of a link between damaged tubules and macrophage activation via SAP130/miR-219c-3p/Mincle axis might provide a new therapeutic potential for necroinflammation in AKI.

## Materials and methods

### Animals

Mincle KO (Mincle^−/−^) mice on the C57BL/6 J genetic background were kindly provided by Dr. Sho Yamasaki (Osaka University, Osaka, Japan) [43]. All animal experiments were approved by the Committee on the Ethics of Animal Experiments of Southeast University. 6–8-week-old male C57BL/6 J mice were purchased from Beijing Vital River Laboratory Animal Technology Co. Ltd.

### Mouse model of kidney injury

Mice were randomly assigned into different groups. A mouse model of unilateral ureteral obstruction (UUO) nephropathy was induced in the male C57BL/6 J mice at 8 weeks of age (20–25 g body weight) by the left ureteral ligation as described previously(*n* = 5–6 for each group) [44]. For cisplatin-induced AKI mouse model, C57BL/6 mice received i.p. cisplatin (Sigma-Aldrich, USA) at a dose of 20 mg/kg as described before and were killed at day 3 (*n* = 5–6 for each group). Lentiviruses expressing miR-219c and nonsense control (NC) constructed in the GVC396 vector were purchased from Genechem (Shanghai, China). Lentiviruses mediated gene transfer in kidneys was performed by intraparenchymal injection (5 × 10^7^TU/mouse). One week post-injection, UUO surgery was performed and animals were killed at day 3 (*n* = 4–5 for each group). To demonstrate the effect of damaged tubules on kidney inflammation, male C57BL/6 J mice (WT and Mincle^-/-^ mice) were administered intraperitoneally with either supernatant from dead TECs (Necrotic medium) at a dose of 5 ml/kg or primed with a low dose of LPS (5 mg/kg, Sigma-Aldrich, USA), and mice were sacrificed 24 h later. To further explore the specific role of SAP130 in renal interstitial inflammation, both WT and Mincle^-/-^ mice were administrated intraperitoneally with a lower dose of LPS (2 mg/kg, Sigma-Aldrich, USA) to induce basic Mincle expression following the application of rSAP130 (H00023450-Q01, Abnova; 5ug per mouse) by tail intravenous injection, and then mice were euthanized 24 h later (*n* = 5 for each group). No blinding was done.

### Acute tubular necrosis (ATN) patients and controls

Patients with biopsy-proven acute tubular necrosis (ATN) (*n* = 9) and glomerular minor lesion (*n* = 8) as controls were enrolled in this study. The study was approved by the Ethics Committee of Zhongda Hospital, Southeast University. Informed consent was obtained from all subjects. All the laboratory measurements, including blood urea nitrogen (BUN), serum creatinine (SCr), uric acid (UA), 24 h proteinuria, were obtained on the day before kidney biopsy. The basic clinic characteristics were present in Table 1. Renal biopsy was used for SAP130 and Mincle immunostaining.

### Cell culture

A mouse macrophage cell line RAW264.7 (ATCC) was used for in vitro study. Raw264.7 macrophages were cultured in RPMI 1640 (Hyclone, USA) media supplemented with 1%(v/v) penicillin-streptomycin (P/S, Gibco) and 10% FBS (0500, Sciencell, USA). mTECs (immortalized mouse tubular epithelial cells) were gift from Dr.Jeffrey B. Kopp, National Institutes of Health. mTECs were cultured in DMEM/F12 (Hyclone, USA) supplemented with 10% fetal bovine serum (Sciencell, USA), 1%(v/v) penicillin, and streptomycin (Gibco, USA).

Supernatant from dead TECs for macrophage treatment were prepared as described before with modification [14, 18]. TECs were heated for 1 h at 52 °C and was incubated for 5 h at 37 °C. Trypan blue staining was used to confirm cell death after thermal injury and detected by stained by Countess™ II FL Automated Cell Counter (Invitrogen, USA). The resulting necrotic cell supernatant were centrifuged at 2000× g for 15 min to eliminate the cell debris and used for experiments after concentration by ultrafiltration (Millipore, MWCO100kD).

### MicroRNA mimics and small interfering RNA (siRNA) transfection

The Lipofectamine™ 2000 (Invitrogen Biotechnology) was used for transfection of miR-219c-3p mimic (sense 5′−3′ CGAGAAUUGCGUUUGGACAAUC, antisense 5′− 3′ UUGUCCAAACGCAAUUCUCGUU) at a concentration of 20 nM according to the manufacturer’s instructions. To knock down SAP130, mTECs were transfected with 50 nM of small, interfering RNA against mouse SAP130 (sense 5′−3′ GGCGUCAGAAUUUGGAAAUTT, antisense 5′− 3′ AUUUCCAAAUUCUGACGCCTT) (designed and synthesized by Shanghai GenePharma Co., Ltd., China). A scramble sense-control was used as negative control (NC).

### Assessment of renal injury

The kidney tissues collected for histology analysis were fixed with 4% formaldehyde and embedded in paraffin for periodic acid-Schiff (PAS) staining. Histopathological changes of renal tubules including loss of brush border, tubular dilation, cast formation, and cell lysis, were evaluated. Renal tubular necrosis was scored semiquantitatively, in which the percentage of cortical tubules showing necrosis was assigned a score of 0, none; 1, 10%; 2, 10–25%; 3, 25–75%; 4, >75% [45].

### Immunohistochemistry and immunofluorescence staining

Antigen retrieval of all paraffin-embedded kidney sections was performed before immuno-staining by microwave heating method in EDTA (MVS-0098, MXB Biotechnologies, Foochow, China) and Citrate antigen retrieval solution (MVS-0100, MXB Biotechnologies, Foochow, China) for kidney sections from mice and patients, respectively. For IHC, formalin-fixed and paraffin-embedded kidney sections were incubated with primary antibodies against F4/80 (ab6640, Abcam, UK) and CD68 (ab201340, Abcam, UK), an ultrasensitive streptavidin peroxidase detection system was used for detection (MXB Biotechnologies, Foochow, China). Quantitative analysis of the number of positive cells was performed under original magnification (×200) in 20 randomly selected fields per mouse in a blinded fashion. Immunofluorescence staining of paraformaldehyde-fixed kidney sections was performed with primary antibody against Mincle (CLEC-4E (B-7): sc-390806, Santa Cruz, USA), SAP130 (sc-398670, Santa Cruz, USA), and CD68 (ab125212, Abcam, UK), followed by incubation with secondary antibodies. Cell nuclei were stained with DAPI. Immunostained samples were visualized under a confocal microscope (FV3000, Olympus).

### Western blotting

Western blotting was performed as previously described [46]. Primary antibodies of Anti-Mincle (D292-3, MBL International, Woburn, MA, USA), anti-iNOS (ab15323, Abcam, UK), anti-GAPDH (CW0100M, CWBio, Beijing, China), anti-β-actin (sc47778, Santa Cruz, USA), anti-SAP130 (sc-398670, Santa Cruz, USA) were used in this study. Secondary HRP-conjugated antibodies (anti-mouse IgG, anti-rabbit IgG, and anti-rat IgG) were used for detection by an ECL advanced system (GE Healthcare). Intensity values expressed as the relative protein expression which were normalized to β-actin or GAPDH, were analyzed by the Image J software.

### Quantitative real-time PCR

The total RNA was extracted from the renal cortex of mice and cultured cells using RNAiso Plus (Vazyme, Nanjing, China), according to the manufacturer’s instructions. Reverse transcription and quantitative renal-time PCR were performed using 5× HiScript III qRT SuperMix and 2× ChamQ SYBR qPCR Master Mix (Vazyme, Nanjing, China). All the data were normalized to GAPDH. All the primers for RT-PCR were listed in Table 2. For quantitative analysis of miR-181a-5p, miR-125a-3p, miR-150-5p, miR-125b-2-3p, and miR-219c-3p, All-in-One™ miRNA qRT-PCR Detection Kit and primers from Genecopeia (Guang Zhou, China) were used.

**Table 2:** Primers of quantitative RT-PCR.

Primer	Forward	Reverse
Mouse MCP-1	CTTCTGGGCCTGCTGTTCA	CCAGCCTACTCATTGGGATCA
Mouse IL-6	AAAGAGTTGTGCAATGGCAATTCT	AAGTGCATCATCGTTGTTCATACA
Mouse TNF-α	CATCTTCTCAAAATTCGAGTGACAA	TGGGAGTAGACAAGGTACAACCC
Mouse IL-1β	TGCCACCTTTTGACAGTGATG	AAGGTCCACGGGAAAGACAC
Mouse GAPDH	GCATGGCCTTCCGTGTTC	GATGTCATCATACTTGGCAGGTTT
Mouse Mincle	ACCAAATCGCCTGCATCC	CACTTGGGAGTTTTTGAAGCATC
Mouse SAP130	ACTACGCCCAGACCCTAACA	AGGAACAATACGGCGACATC
Mouse miR-219c-3p	CGAGAAUUGCGUUUGGACAAUC	

### Luciferase reporter assay

The miR-219c-3p overexpression GV251-vector and the clec4e-3′-UTR luciferase reporter GV272 vector were constructed. To test the binding specificity, we mutated the sequences in the clec4e3′-UTR that interact with the miR-219c-3p seed sequence (from “AATTCTC” to “CCGGAGA”). 293 T cells were cotransfected in 24-well plates with 0.1 μg of 3′ UTR Luciferase vector, 0.4 μg of miRNA vector and 0.02ug of the control vector containing Renilla luciferase. Firefly and Renilla luciferase activities were quantified in lysates using the Dual Luciferase Reporter Assay kit (Promega) according to the manufacturer’s recommendations.

### Statistical analysis

Data are expressed as mean ± standard deviation (SD) or as mean ± standard error of mean (S.E.M) of each group. A two-tailed unpaired Student’s *t*-test or Mann–Whitney U test was used for comparison between two groups, and one-way ANOVA was performed for comparisons of data with more than two groups for multiple comparisons. All analyses were carried out by GraphPad Prism 7.0. *P* < 0.05 was considered statistically significant.

## Data Availability

All data generated or analyzed during this study are included in this published article.
